# The oral bioaccessibility and gingival cytotoxicity of metal(loid)s in wild vegetables from mining areas: Implication for human oral health

**DOI:** 10.3389/fnut.2022.1042300

**Published:** 2022-11-04

**Authors:** Wen Tian, Peng Gao, Da-Peng Zong, Jian-Jun Liu, Meng-Yan Zhang, Cheng-Chen Wang, Zhen-Xing Wang, Jian-Min Wang, You-Ya Niu, Ping Xiang

**Affiliations:** ^1^Yunnan Province Innovative Research Team of Environmental Pollution, Food Safety, and Human Health, School of Ecology and Environment, Southwest Forestry University, Kunming, China; ^2^Department of Environmental and Occupational Health, University of Pittsburgh School of Public Health, Pittsburgh, PA, United States; ^3^Yunnan Key Laboratory of Stem Cell and Regenerative Medicine, Institute of Biomedical Engineering, Kunming Medical University, Kunming, China; ^4^School of Life Science, Southwest Forestry University, Kunming, China; ^5^Yunnan Rural Science and Technology Service Centre, Kunming, China; ^6^School of Basic Medical Sciences, Hunan University of Medicine, Huaihua, China

**Keywords:** wild vegetables, heavy metal(loid)s, oral bioaccessibility, gingival cytotoxicity, health risk

## Abstract

**Background:**

Heavy metal(loid)s are frequently detected in vegetables posing potential human health risks, especially for those grown around mining areas. However, the oral bioaccessibility and gingival cytotoxicity of heavy metals in wild vegetables remain unclear.

**Methods:**

In this study, we assessed the total and bioaccessible Cr, As, Cd, Pb, and Ni in four wild vegetables from mining areas in Southwest China. In addition, the cytotoxicity and underlying mechanisms of vegetable saliva extracts on human gingival epithelial cells (HGEC) were studied.

**Results:**

The *Plantago asiatica L*. (*PAL*) showed the highest bioaccessible Cr, As, Cd, and Pb, while the greatest bioaccessible Ni was in *Taraxacum mongolicum* (*TMM*). The *Pteridium aquilinum* (*PAM*), *Chenopodium album L*. (*CAL*), and *TMM* extracts decreased cell viability, induced apoptosis, caused DNA damage, and disrupted associated gene expressions. However, *PAL* extracts which have the highest bioaccessible heavy metals did not present adverse effects on HGEC, which may be due to its inhibition of apoptosis by upregulating *p53* and *Bcl-2*.

**Conclusion:**

Our results indicated that polluted vegetable intake caused toxic effects on human gingiva. The heavy metals in vegetables were not positively related to human health risks. Collectively, both bioaccessibility and toxic data should be considered for accurate risk assessment.

## Introduction

Heavy metal(loid)s are wildly distributed in the environment, such as soil, and plants (1). Anthropogenic activities are the main contributors to heavy metal(loid)s in the environment (2). Smelting and mining operations are the most important anthropogenic sources of heavy metal(loid)s. Mining of polymetallic ores associated with sulfide minerals, lead–zinc, and copper can release Cr, As, Cd, Pb, and Ni into the environment (3, 4), which may result in their accumulation in the surrounding plants such as wild vegetables.

Wild vegetables are uncultivated but edible plants and distributed around the world. By supplying the body with protein, minerals, vitamins, and certain hormone precursors, wild vegetables play an important role in the human daily diet, especially in some developing countries such as China, Russia, and Thailand (5). Also, some of them provide important health benefits because of their nutritional and pharmacological characteristics (6). *Plantago asiatica L*. and *Taraxacum mongolicum* are traditional medicinal and representative wild vegetables in China for their variously beneficial functions, such as anti-oxidative, anti-inflammatory, and anti-microbial properties. In addition, *Pteridium aquilinum* and *Chenopodium album L*. are popular wild vegetables as well due to affluent nutrients such as polyunsaturated fatty acids, vitamin C, and carotene (7–9). Since the wild vegetables are uncultivated by humans and the heavy metal(loid)s in them are not regulated and monitored, consuming the wild vegetables that grew up on contaminated sites would lead to human health risks (4). In addition, the bioaccessibility of heavy metal(loid)s was employed to accurately assess exposure health risks as an *in vitro* assay by predicting the *in vivo* bioavailable fractions of the heavy metal(loid)s (10, 11).

Among exposure pathways, oral ingestion is an important contributor. The oral cavity is the original phase of the alimentary system, which contains various organs, such as teeth, tongue, and salivary glands. Ingesting heavy-metal(loid)-contaminated foods could lead to acute toxicities or chronic accumulations in human tissues and contribute to certain diseases (12). For instance, periodontitis is a representative inflammatory disease in the gingival tissues (13). Gingival epithelial cells play important roles in maintaining oral immunologic homeostasis and protecting oral organs from inflammatory damage (14). Ingestion of heavy-metal(loid)-contaminated wild vegetables may expose gingival epithelial cells to heavy metal(loid)s, especially during the chewing procedure. As such, the adverse effects of heavy metal(loid)s on human gingival epithelial cells and the underlying molecular mechanisms are still unclear. However, heavy metal(loid)s have been demonstrated to cause cytotoxicity, induce apoptosis and DNA damage, and alter related genes expressions (e.g., *Caspase-3, p53*, and *GADD45*α) to human oral mucosa cells, gastric adenocarcinoma cells, and colonic epithelial cells (15–17).

As one of the known vegetable kingdoms, Yunnan has abundant plant resources such as wild vegetables. Residents consume wild vegetables purchased from markets or collected by themselves, especially in spring. To evaluate the oral health risk of heavy metal(loid)s consumption of wild vegetables from mining areas, in this study, we investigated the pollution levels of Cr, As, Cd, Pb, and Ni in four common edible wild vegetables as well as their corresponding growing soils from mining areas in Yunnan, Southwest China. Also, the toxic effects of wild vegetable extracts on human gingival epithelial cells were assessed by analyzing cell morphology, viability, apoptosis, intracellular DNA damage, and the expressions of related genes. Overall, this study aims to provide a refining health risk assessment method of the wild vegetable extracts including heavy metal(loid)s on human gingiva *via* ingestion exposure.

## Materials and methods

### Study area and sampling

**Figure 1:**
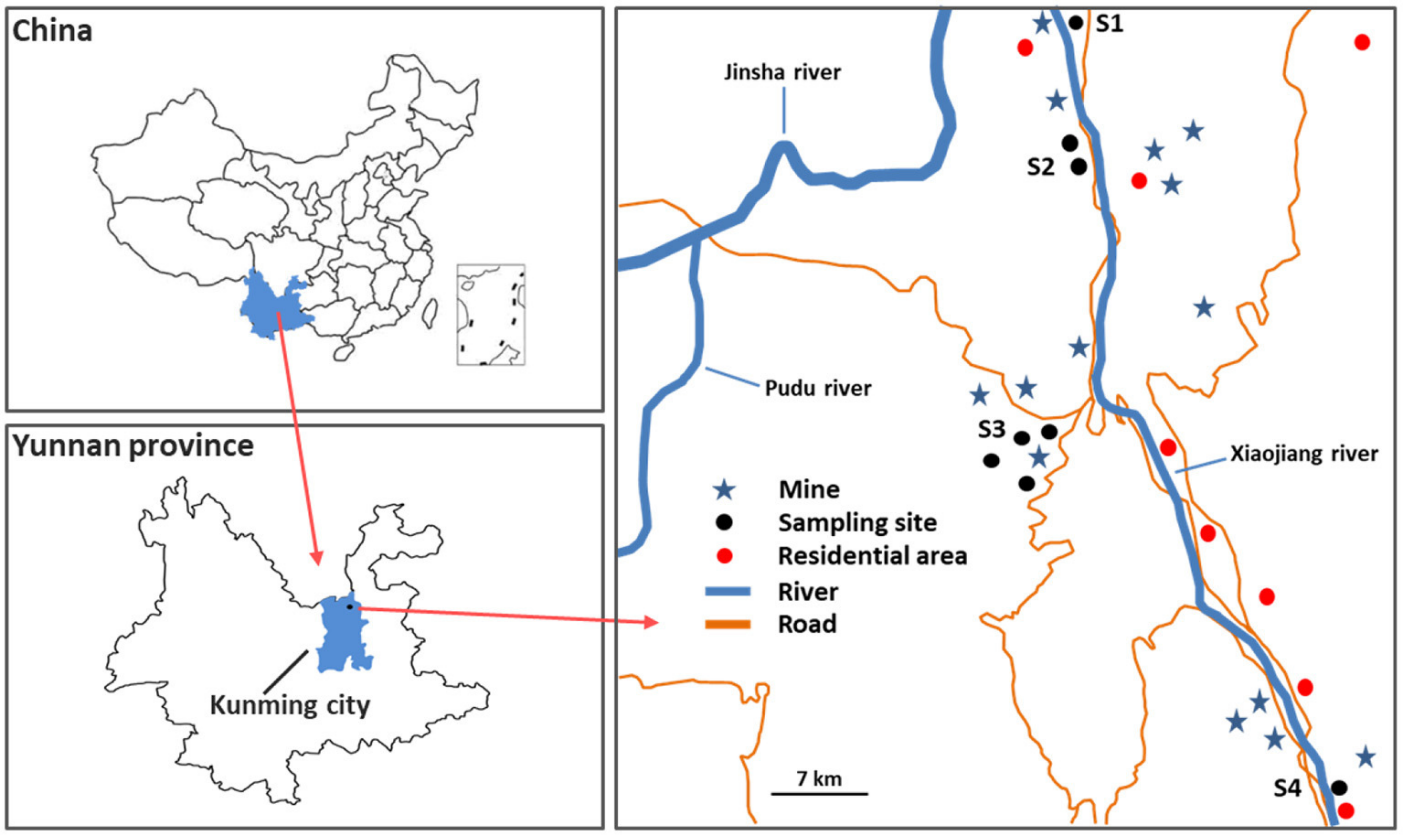
Schematic display of study area and sampling sites.

The study area is an industrial district in the central region of Yunnan Province, Southwest China, which as a site for Cu mining and smelting (Figure 1), it is influenced by a subtropical plateau monsoon climate, holding an annual average temperature of 21.2°C, the average annual precipitation is 560.9 mm with a relative humidity of 76%. The rainy season comes in May every year, the plants will have a better living environment since then. The Cu mining and smelting sites had more than 2,000 years, which caused intermittent water and soil loss, along with discharged/emitted wastewater, solid wastes, and dust. Heavy metal(loid)s would be accumulated in soils and plants surrounding the mines. However, there are few studies on the pollution status and health risk of heavy metal(loid)s in local wild edible vegetables.

Four sampling sites (S1–S4) were selected in the vicinity of the mines in Xiaojiang River Basin, Dongchuan District, Kunming city, Yunnan province, Southwest China. And four common types of local wild edible vegetables (eight samples) including *Pteridium aquilinum* (*PAM*), *Plantago asiatica L*. (*PAL*), *Chenopodium album L*. (*CAL*), and *Taraxacum mongolicum* (*TMM*), as well as their corresponding growing soils (eight samples) were collected from S1–S4 in June 2021. All wild vegetable samples were washed three times with distilled water and dried to constant weight at 75°C, then crushed with a porcelain mortar and pestle, and kept in a refrigerator before analyses. All soil samples were air-dried and grounded to pass through a 100-mesh nylon sieve before analyses.

### Measurement of metal(loid)s contents

The total contents of heavy metal(loid)s in soils were analyzed by an X-ray fluorescence spectrometer (XRF, E-max 500). Due to XRF with a high limit of detection, the total and bioaccessible concentrations of heavy metal(loid)s in wild vegetables were determined by inductively coupled plasma mass spectrometry (ICP-MS, ICAPQR, Thermo Fisher Scientific, USA) (18). For total contents, wild vegetable samples (0.10 g, dry weight) were digested with concentrated HNO_3_ (Guaranteed Reagent) and 30% H_2_O_2_ (Guaranteed Reagent), then analyzed by ICP-MS. Based on total concentrations of heavy metal(loid)s in all wild vegetables, we selected four wild vegetable samples for the following experiments.

Bioaccessible heavy metal(loid)s in wild vegetables were extracted using artificial saliva (pH = 7, Fusayama, Shanghai Lianshuo Biological Technology, China) (19). The compositions of the artificial saliva include NaCl, KCl, CaCl_2_, NaH_2_PO_4_, and urea. Briefly, wild vegetable samples (0.50 g, dry weight) were dissolved in 20 mL artificial saliva and extracted at 150 rpm and 37°C in an oscillator for 24 h. After that, each extract was centrifuged and the supernatant was passed through a 0.22 μm sterile syringe filter (Corning, NY, USA), and collected in two sterile tubes, respectively. The content of the supernatant in one tube was applied for ICP-MS analysis, while the supernatant in another tube was used for cell experiments.

### Cell culture and exposure

Human gingival epithelial cells (HGEC) were purchased from the American type culture collection (Manassas, VA, USA). HGEC were grown in Dulbecco's Modified Eagle Medium (DMEM) supplemented with 10% fetal bovine serum (FBS) and 1% penicillin-streptomycin (PS) in a humidified atmosphere with 5% CO_2_ at 37°C, and the media was substituted with fresh medium every alternate day.

Before each exposure, the logarithmic growth phase HGEC was trypsinized and reseeded into 6/24/96-well plates overnight to allow cell attachment for use. Each wild vegetable extract was mixed with DMEM 1:4 to obtain the exposure solution. The diluted extracts were added to the 6/24/96-well plates and incubated for 24 h, with a blank artificial saliva solution as the control.

### Cytotoxicity and apoptosis assay

To determine the impacts of extracts on HGEC proliferation, HGEC were cultured in a 96-well plate after overnight incubation at the density of 8 × 10^3^ cells/100 μL/well. After exposure to the extracts for 24 h, cellular morphology was recorded by an inverted microscope (TS-100, Nikon, Japan). Simultaneously, a cell counting kit-8 assay kit (CCK-8, Yi Fei Xue Biotechnology, China) was employed to analyse cell proliferation. To calculate cell viability, the absorbance was measured at 450 nm with a microplate reader (Molecular Devices LLC, USA).

Annexin V-FITC/PI apoptosis assay kit was used for cell apoptosis analysis. In brief, HGEC were seeded in 6-well plates (1 × 10^6^ cells per well) with DMEM for 24 h. After that, the spent DMEM was replaced with the exposure solution and treated for 24 h. After the exposures, cells were harvested and washed with cold phosphate buffer saline (PBS) three times and then immersed in 500 μL of PBS binding buffer (containing 5 μL Annexin V-FITC and 5 μL of PI-staining solution) for 15 min at room temperature in the dark. Approximately 1 × 10^4^ cells were loaded with a CyFlow^®^ Cube 6 Flow cytometer (Sysmex Partec, Germany) to assess cell apoptosis, and then analyzed by Flow Jo Version 10.0.6 software (BD Biosciences, USA).

### DNA damage assay

Direct immunofluorescence staining was employed to assay the DNA damage of HGEC induced by extracts. Briefly, HGEC were replated into 24-well plates at 5 × 10^4^ cells/well for 1 day and then exposed to extracts for 24 h at 37°C. Cells were rinsed three times with PBS, fixed by 4% poly-formaldehyde for 30 min, and permeabilized by 10% Triton X-100 for 15 min. After being washed three times with PBS, cells were blocked with 1% bovine serum albumin (BSA) for 60 min. Subsequently, cells were incubated overnight at 4°C with the rabbit monoclonal antibody anti-γ-H2AX (ab81299, Abcam, USA). After being maintained at room temperature for 1 h, cells were immersed in PBS for 5 min, then incubated by Goat Anti-Rabbit IgG (H+L) Fluor488-conjugated (Affinity Biosciences, USA) at room temperature for another 1 h. Last, the cells had a 10 min dark incubation at 37°C with 4', 6-diamidino-2-phenylindole (DAPI, Service bio, China) for nuclear counterstaining. Immunofluorescent images were observed and photographed with an inverted microscope system (IX73, Olympus, Japan).

### Gene expressions analyses

To further investigate the mechanisms of cell apoptosis and DNA damage, the associated regulatory gene expression levels were estimated. After each exposure, the total RNA of HGEC was extracted using the total RNA isolation kit (Yi Fei Xue Biotechnology, China). The concentration and quality of RNA were determined using a UV–Vis Spectrophotometer (Q5000, Quawell, USA). The cDNA was synthesized from total RNA by the commercial reverse transcription kit. The quantitative real-time polymerase chain reaction (qRT-PCR) was performed to detect related gene expression levels (*Bax, Bcl-2, p53, p21, GADD45*α, *Caspase-3, Caspase-8*, and *Caspase-9*) by applying SYBR Green qPCR Master Mix and Roche LightCycler 480II Real-Time PCR system (Roche, Swiss). The reaction cycle condition started at 95°C for 15 min, followed by 40 cycles for 15 s, then maintained at 60°C for 1 min, and β*-Actin* was utilized as an internal reference. The data were calculated by the 2^−Δ*ΔC*^T method (20), with the primer sequences being listed in Table 1.

**Table 1:** Primers for qRT-PCR of cell apoptosis regulatory genes.

**Gene**	**Forward primer (5^′^–3^′^)**	**Reserve primer (5^′^–3^′^)**	**Accession no**.	**Production size (bp)**
*Bax*	CCCGAGAGGTCTTTTTCCGAG	CCAGCCCATGATGGTTCTGAT	NM_138761.3	155
*Bcl-2*	GGTGGGGTCATGTGTGTGG	CGGTTCAGGTACTCAGTCATCC	NM_000633.2	89
*p53*	CAGCACATGACGGAGGTTGT	TCATCCAAATACTCCACACGC	NM_000546.5	125
*p21*	TGTCCGTCAGAACCCATGC	AAAGTCGAAGTTCCATCGCTC	NM_001291549.1	139
*GADD45α*	GAGAGCAGAAGACCGAAAGGA	CAGTGATCGTGCGCTGACT	NM_001924	87
*Caspase-3*	CATGGAAGCGAATCAATGGACT	CTGTACCAGACCGAGATGTCA	NM_004346.3	139
*Caspase-8*	CGGACTCTCCAAGAGAACAGG	TCAAAGGTCGTGGTCAAAGCC	NM_033355.3	199
*Caspase-9*	CTCAGACCAGAGATTCGCAAAC	GCATTTCCCCTCAAACTCTCAA	NM_032996	116
*β-Actin*	GTACCACTGGCATCGTGATGGACT	CCGCTCATTGCCAATGGTGAT	NM_001101.3	323

### QA/QC and statistical analysis

Reagent blank, artificial saliva solution, and certified reference materials (GBW07405 and GBW10015a, Institute of Geophysical & Geochemical Exploration, China) were used to ensure quality control and assurance. The recovery rates were all above 80%. All the samples had triplicate determinations to ensure precise results. The accuracy of the measurements is assured within 10% RSD. ICP-MS detection limits of Cr, As, Cd, Pb, and Ni are 0.0002, 0.0002, 0.0003, 0.0001, and 0.002 mg/kg, respectively. XRF detection limits of Cr, As, Cd, Pb, and Ni are 5, 0.2, 0.05, 0.8, and 1 mg/kg, respectively. Statistical analysis was conducted by GraphPad Prism Version 8.0 software (GraphPad Software LLC, CA, USA). Data are presented as the mean ± standard deviation, and differences among exposure groups and the control group were analyzed by one-way ANOVA at *p* < 0.05.

## Results and discussion

### Contents of heavy metal(loid)s in wild vegetables

**Figure 2:**
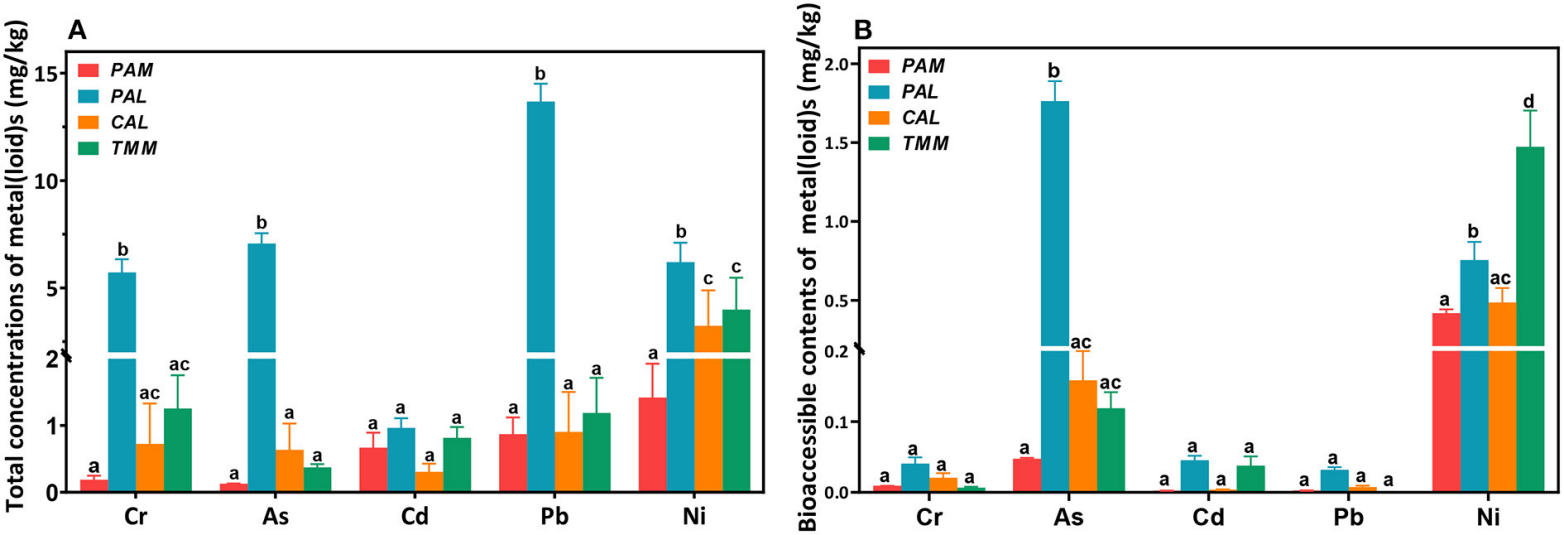
Total **(A)** and bioaccessible **(B)** concentrations of heavy metal(loid)s in wild vegetables. Error bars represent the mean ± SD of values from triplicate experiments. Different letters indicate significant differences (*p* < 0.05).

Total concentrations of Cr, As, Cd, Pb, and Ni (mg/kg, dry weight) in the wild vegetables were shown in Figure 2A. Generally, Ni or Pb had the highest concentrations among all heavy metal(loid)s in *PAL* (13.7 ± 0.83 mg/kg), *TMM* (3.99 ± 1.49 mg/kg), *CAL* (3.24 ± 1.65 mg/kg), and *PAM* (1.42 ± 0.51 mg/kg). Interestingly, Pearson correlation analyses indicated that Ni contents in wild vegetables were negatively correlated with those in soils (*r* = −0.99, *p* < 0.05). These results were consistent with the concentrations found in cabbages that grew up in the same areas, and atmospheric deposition of the sampling site probably increased the absorption of metal(loid)s in the overground part of wild vegetables, which affected the results (21, 22). Cr, As, and Cd revealed varied levels in different wild vegetables, with the lowest concentrations of Cr (0.19 ± 0.06 mg/kg) and As (0.13 ± 0.00 mg/kg) found in *PAM*, and Cd (0.31 ± 0.12 mg/kg) in *CAL*. The results also showed lower levels of Cr, Cd, and Pb in *TMM* compared with the previous study (23), which could be explained by the fact that lower contamination levels of the corresponding soils in the current study (Table 2). In addition, *PAL* was contaminated by the highest levels of Cr, As, Cd, Pb, and Ni among four wild vegetables, with average concentrations being 5.72 ± 0.61, 7.07 ± 0.48, 0.96 ± 0.14, 13.7 ± 0.83, and 6.20 ± 0.90 mg/kg, respectively. Cr and Cd levels were lower when compared with Abbasi et al. (6) study, but Pb was higher in our study, which may be attributed to the different industrial activities. In addition, *PAL* corresponding soil did not have the highest concentrations of metal(loid)s compared with the other three soils (Table 2), indicating that *PAL* easily accumulated the above-mentioned metal(loid)s, especially for As, which is consistent with previous studies (24, 25). Although the concentrations of As, Cd, Pb, and Ni in *PAM* corresponding soils were higher compared to the other three vegetable soils (Table 2), the levels of heavy metal(loid)s in *PAM* were lower than those in the others. The differences in metal(loid)s contents of wild vegetables and their corresponding soils may be caused by the different contents of metal(loid) in environmental matrices as well as uptake capacities and preferences of various plant species (2, 24, 26). Furthermore, bioaccumulation coefficients of metal(loid)s, soil physical and chemical characteristics, and environmental parameters also play important roles in the transportation and accumulation of heavy metal(loid)s from soils to plants (1, 27). In addition, the average concentrations of heavy metal(loid)s in wild vegetables exceeded the leafy vegetables regulatory limit (GB2762−2017, China; WHO/FAO, 2019).

**Table 2:** Total concentrations (mg/kg) of heavy metal(loid)s^a^ in wild vegetables and their corresponding growing soils.

**Site**	**Sample**	**Wild vegetable**					**Soil**				
		**Cr**	**As**	**Cd**	**Pb**	**Ni**	**Cr**	**As**	**Cd**	**Pb**	**Ni**
S3	*TMM-1*	0.64 ± 0.36	0.88 ± 0.55	0.59 ± 0.19	2.08 ± 0.08	2.27 ± 0.39	83.63 ± 5.31	50.3 ± 4.25	1.83 ± 0.09	221.9 ± 9.94	69.30 ± 1.84
S3	*PAM*	0.19 ± 0.06	0.13 ± 0.00	0.67 ± 0.22	0.87 ± 0.25	1.42 ± 0.51	49.45 ± 0.76	115.7 ± 1.23	4.48 ± 0.03	193.4 ± 0.30	90.17 ± 1.26
S3	*CAL-1*	2.02 ± 0.59	1.07 ± 0.19	2.49 ± 0.37	5.60 ± 0.36	1.71 ± 0.05	66.40 ± 1.25	39.7 ± 0.96	1.73 ± 0.04	149.1 ± 0.78	62.25 ± 0.38
S2	*TMM-2*	4.02 ± 0.81	2.59 ± 0.49	0.97 ± 0.06	3.97 ± 0.47	2.51 ± 0.43	34.90 ± 1.17	20.9 ± 0.72	0.96 ± 0.01	26.23 ± 0.49	26.70 ± 1.51
S2	*PAL*	5.72 ± 0.61	7.07 ± 0.48	0.96 ± 0.14	13.7 ± 0.83	6.20 ± 0.90	117.8 ± 5.65	7.30 ± 0.60	0.54 ± 0.01	34.90 ± 0.60	11.25 ± 0.05
S1	*CAL-2*	1.04 ± 0.61	2.45 ± 0.16	0.43 ± 0.29	2.10 ± 1.02	2.55 ± 0.15	59.40 ± 3.35	52.5 ± 3.86	2.11 ± 0.25	50.73 ± 2.78	88.87 ± 3.63
S4	*CAL*	0.72 ± 0.61	0.64 ± 0.39	0.31 ± 0.12	0.90 ± 0.60	3.24 ± 1.65	161.9 ± 5.64	8.31 ± 0.02	0.72 ± 0.03	12.40 ± 1.97	62.50 ± 1.32
S3	*TMM*	1.26 ± 0.50	0.37 ± 0.05	0.82 ± 0.16	1.19 ± 0.53	3.99 ± 1.49	130.2 ± 5.05	25.5 ± 2.48	1.09 ± 0.17	57.40 ± 2.95	51.15 ± 1.15
Regulatory limit ^b^/		0.5	0.5	0.2	0.3	–^d^	65.20	18.40	0.218	40.60	42.50
background value^c^											

a The results were presented in mean value ± SD (standard deviations).

b Regulatory limits of Cr, As, Cd, and Pb in vegetables recommended by the WHO/China are 0.5, 0.5, 0.2, and 0.3 mg/kg in leafy vegetables, respectively.

c Background value: soil background value of Yunnan province, southwest China.

d Not given.

Bioaccessible concentrations of Cr, As, Cd, Pb, and Ni in wild vegetables were presented in Figure 2B. Among all the wild vegetables, the highest bioaccessible concentrations of Cr (0.04 ± 0.008 mg/kg), As (1.76 ± 0.13 mg/kg), Cd (0.045 ± 0.006 mg/kg), and Pb (0.03 ± 0.004 mg/kg) were found in *PAL*, consistent with the results of total concentrations. As a bioremediation plant, *PAL* can accumulate various heavy metal(loid)s (24). The highest bioaccessible Ni (1.47 ± 0.23 mg/kg) was found in *TMM*, which is consistent with a previous study (7). Especially, the bioaccessible Pb in *PAL* was reduced to 0.032 ± 0.003 mg/kg compared with its total concentration of 13.7 ± 0.83 mg/kg, similar to the other study (28). Since inorganic Pb (II) is the main lead speciation in food (29) and it is barely soluble in alkaline solutions, the soluble fraction of Pb in extracts (pH = 7.5–9.3) should be low. Although the bioaccessible concentrations of heavy metal(loid)s in wild vegetables were much lower than the total concentrations, we further conducted cytotoxicity experiments to investigate whether these bioaccessible extracts have adverse effects on HGEC.

### Wild vegetable extracts induced cytotoxicity

**Figure 3:**
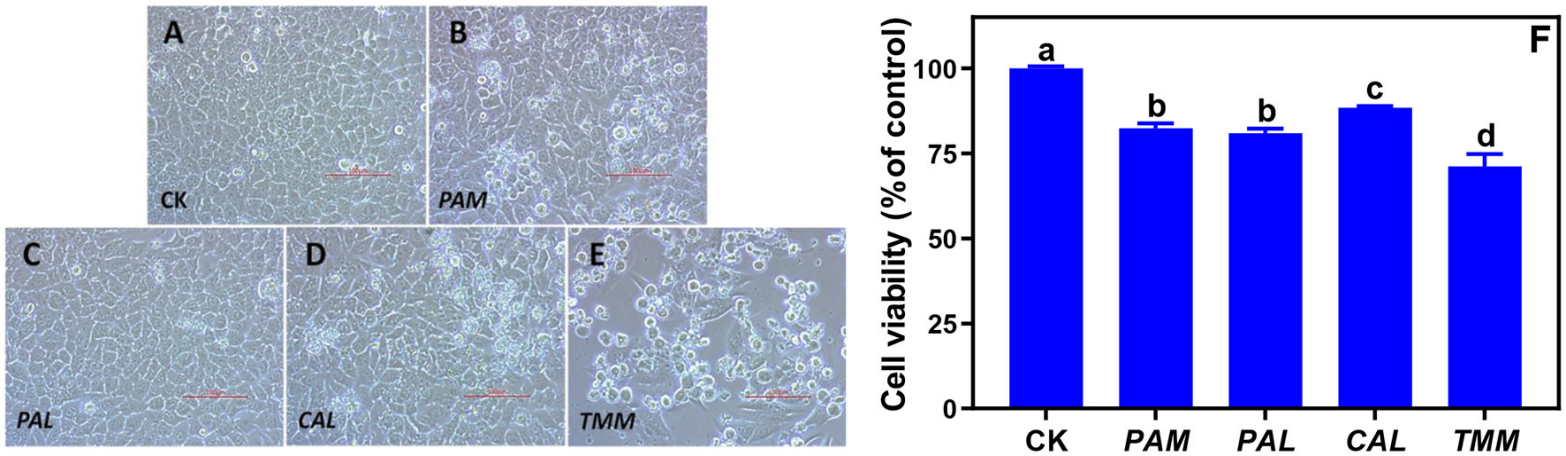
Alterations of HGEC morphology and viability after being exposed to the extracts of wild vegetables for 24 h. Normal HGEC morphology in control (**A**; CK) and altered cell morphology and viability after exposures to the extracts of *PAM* **(B)**, *PAL* **(C)**, *CAL* **(D)**, and *TMM* **(E)**. Cell viability was determined by the CCK-8 assay kit and was normalized as the percentage of control **(F)**. Typical cobblestone, round, or floated appearances of HGEC were clear in the control group and exposure groups **(A–E)** at 200 × magnification (bar = 100 μm). Error bars represent the mean ± SD of values from triplicate experiments. Different letters indicate significant differences (*p* < 0.05).

The observations of cell morphology and viability are significant visualized indicators to evaluate cytotoxicity, which were widely employed to assay cell survival and proliferation (16, 30). Nevertheless, the impacts of bioaccessible metal(loid)s in vegetables on the morphology and viability of human gingival cells were not reported. Our previous study indicated gastric bioaccessible metals in cabbage did not cause the alteration of cell viability and morphology in human gastric adenocarcinoma cells (31). In this study, transformed cell morphologies were observed under all exposures excluded *PAL*, and cell viabilities were decreased across all exposures (Figures 3A–F). Specifically, HGEC exhibited varied levels of cell viability inhibition from 12 to 29% after being exposed to the extracts, especially after being exposed to *TMM* extracts. *TMM* extracts had the highest bioaccessible concentration of Ni among all vegetables (Figure 2B), and Ni was demonstrated to decrease cell viability and elicit cytotoxicity in oral mucosa cells and gingival fibroblasts (15, 32). Consistently, bioaccessible Ni was negatively correlated with the cell viability (*r* = −0.92, *p* < 0.05) in this study among all vegetables (Table 3).

**Table 3:** Pearson correlation coefficients between wild vegetable bioaccessible metal(loid)s and various toxicity indexes (cell viability, apoptosis, and genes) in HGEC after exposures to four extracts for 24 h.

	**Cr**	**As**	**Cd**	**Pb**	**Ni**
Cell viability	0.36	0.03	−0.66	0.22	–**0.92**^**a**^
Cell apoptosis	−0.03	−0.38	−0.62	−0.22	−0.42
**Genes**					
*Bax*	0.12	0.23	−0.16	0.24	−0.52
*Bcl-2*	0.80	**0.90**	0.47	0.89	−0.26
*Caspase-8*	−0.06	0.03	−0.31	0.04	−0.54
*Caspase-9*	0.16	0.25	−0.18	0.27	−0.56
*Caspase-3*	−0.23	−0.17	−0.48	−0.15	−0.56
*GADD45α*	−0.18	−0.48	–**0.96**	−0.31	−0.84
*p21*	0.35	0.41	−0.13	0.44	−0.63
*p53*	0.89	**0.99**	0.64	**0.96**	−0.10

a The absolute values of coefficients >0.9 between bioaccessible metal(loid)s and various toxicity indexes are bold.

The changes in cellular morphology were consistent with the results of cell viability besides the exposure to *PAL* extract (Figures 3A–E). As one of the most important traditional Chinese herbs, *PAL* is rich in beneficial nutrients such as *PAL* polysaccharides, which could alleviate the cytotoxicity caused by environmental pollutants (8). We speculated *PAL* polysaccharides play an important role in mediating the extract-induced cytotoxicity of *PAL*. Specifically, the typical cobblestone and polygonal appearances of confluent monolayers were clear in the control group (Figure 3A) and the *PAL* exposure group (Figure 3C). However, HGEC emerged with loose and irregular morphologies along with increased floated and round cells under the exposures of *PAM, CAL*, and *TMM* extracts (Figures 3B,D,E), revealing cell deaths. Since these results cannot firmly confirm that bioaccessible heavy metal(loid)s are the causes of cell death, further experiments are crucial to elucidate the mechanisms of the observed cytotoxicity.

### Wild vegetable extracts triggered cell apoptosis

**Figure 4:**
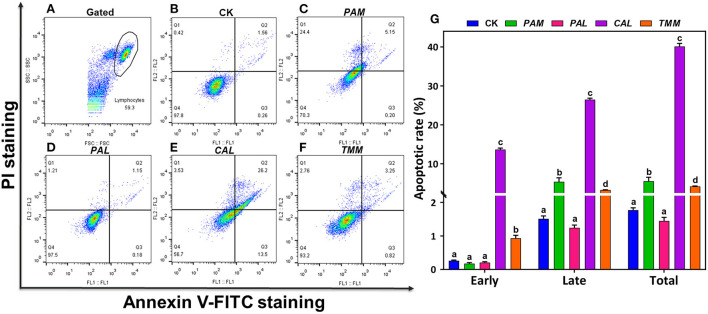
Apoptosis of HGEC after being exposed to extracts for 24 h. FL1 represents Annexin V-FITC staining and FL2 represents PI staining. HGEC populations (**A**; black circle) and apoptosis in the control group (**B**; CK). Apoptosis of HGEC after being exposed to *PAM* **(C)**, *PAL* **(D)**, *CAL* **(E)**, and *TMM* **(F)** extracts: necrotic cells were shown in the Q1 quadrant, viable (late) apoptotic cells and non-viable (early) apoptotic cells were distributed in Q2 and Q3 quadrant, Q4 quadrant was living cells. The statistical graph showed cell apoptosis rate under different treatments (Q2 + Q3 quadrant) **(G)**. Data were presented in mean ± SD. The different letters indicate significant differences (*p* < 0.05).

As a gene-controlled cell death under stress conditions, apoptosis plays an indispensable role in maintaining tissue homeostasis by eliminating cells, but disordered apoptosis induced by xenobiotics is a pathological phenomenon (33). Apoptosis of HGEC after being exposed to extracts for 24 h are shown in (Figure 4). In this study, Annexin V-FITC/PI double staining tests detected cell apoptosis after exposing HGEC to *PAM, CAL*, and *TMM* extracts (Figures 4C,E,F), which was consistent with the corresponding decreases in cell viability (Figure 3F) and changes in cell morphology (Figures 3B,D,E). Similarly, studies reported that heavy metal(loid)s in environmental matrices (e.g., dust, air particulate matter, and foods) caused cytotoxicity and induced apoptosis of human cell lines (34–36). Furthermore, the most elevated apoptotic ratio (39.7%) was observed under the exposure of *CAL* extract compared with the control group (1.82%), implicating *CAL* extract was more potent to induce cytotoxicity and apoptosis than that of other extracts (37). However, the *PAL* extract, which had the highest bioaccessible Cr, As, Cd, and Pb, did not decrease cell viability, change cell morphology, and induce cell apoptosis. It is possibly due to the protective effects of *PAL* polysaccharides, such as alleviating the cytotoxicity caused by environmental pollutants and inducing the maturation of dendritic cells (8, 38). Thus, we speculated *PAL* polysaccharides extracted by the artificial saliva may play pivotal roles in inhibiting *PAL*-extract-induced apoptosis. These results indicated the nutrients in the vegetables may also influence the contaminant-induced toxicity, thus the human health risk assessments simply based on the total concentrations of heavy metal(loid)s may not be accurate enough (39).

### Wild vegetable extracts caused DNA damage

To further elucidate how extracts triggered HGEC apoptosis, a DNA damage assay was performed (40). The damage and repair of DNA play important roles in cell transformation and cell death in the human oral cavity (41), while DNA damage as a blockage in DNA replication could lead to cell apoptosis (42). Histone H2AX phosphorylation on serine 139 is an early signaling event triggered by DNA double-strand breaks. Thus, as the phosphorylated version of histone H2AX, γ-H2AX is a sensitive marker for DNA damage (43).

**Figure 5:**
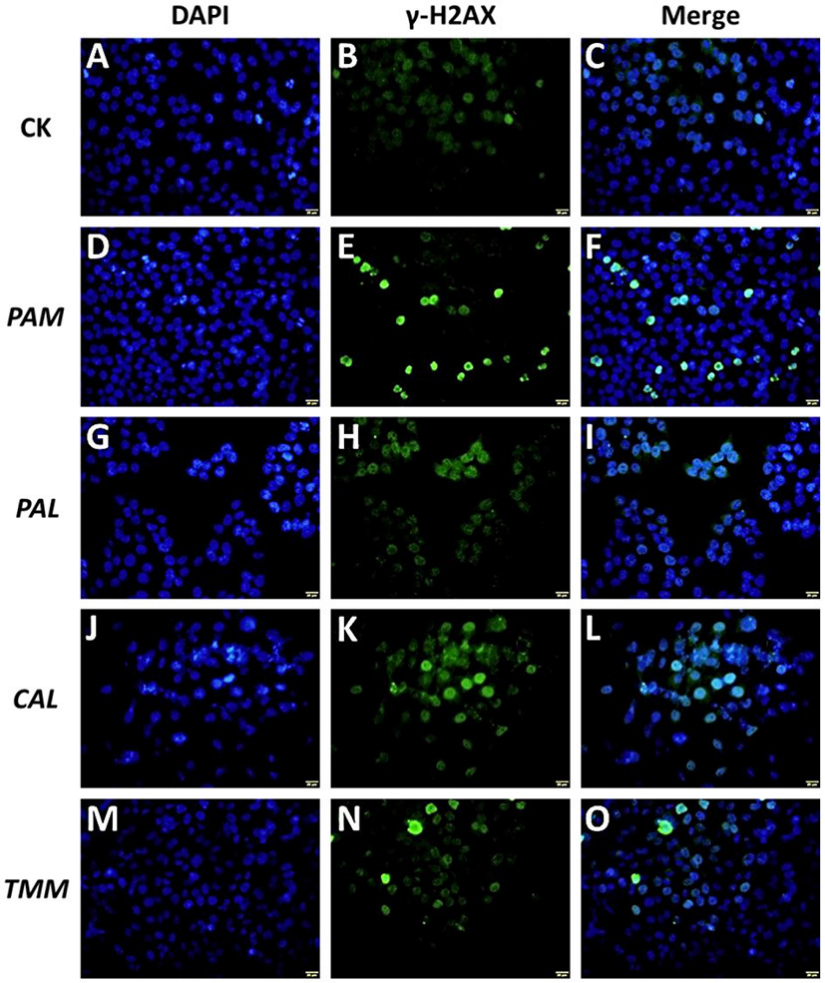
Effects of extracts (*PAM*: **D–F**, *PAL*: **G–I**, *CAL*: **J–L**, and *TMM*: **M–O**) on γ-H2AX protein in HGEC after being exposed for 24 h. Background control (CK) was artificial saliva alone **(A–C)**. The immunofluorescent photographs of the phosphorylation of histone H2AX in γ-H2AX-stained HGEC (Green: **B,E,H,K,N**) and the counterstained nuclei with DAPI (Blue: **A,D,G,J,M**) were captured. Blue dots were cell nuclei, green dots indicated DNA damage. The magnification was 60 × and the scale bar (**A–O**: yellow, located in the lower right corner) is 20 μm.

To further confirm our hypothesis that the observed HGEC apoptosis was caused by extract-induced DNA damage, we assessed DNA damages after the exposures to extracts by using immunofluorescence staining (Figure 5). The results showed that the exposure groups of *PAM, CAL*, and *TMM* extracts presented more H2AX phosphorylation focal points (Figures 5C,F,L,O) compared with that in the control group, while the level of DNA damages in the *PAL* exposure group and control group are similar (Figures 5C,I). These results indicated the extracts of *PAM, CAL*, and *TMM* induced DNA damage in HGEC, which is consistent with the results of cell apoptosis (Figure 4G), morphology (Figures 3B,D,E), and viability (Figure 3F).

Moreover, *PAM* and *CAL* extracts induced more severe DNA damage compared with *TMM* extract (Figures 5F,L,O), even though *TMM* extract had higher bioaccessible Ni (Figure 2B). Although a previous study showed that Ni did not induce DNA damage in gingival cells (44), Hafez et al. (32) found that DNA damages of oral mucosa cells were correlated with the cellular Ni concentrations. Ni subsulfide and nickel oxides had also been demonstrated to induce DNA damage (45). In this study, the extracts with high Ni contents (exclude *PAL*) only slightly exhibited DNA damage on HGEC (Figures 2B, 5F,L,O). These inconsistent results could be attributed to the differences in cellular physiology as well as the constituents of the exposure and exposed systems (46, 47). As such, these comparisons further demonstrated that the cell apoptosis and DNA damages may not be correlated with the levels of heavy metal(loid)s in edible vegetables, but are also impacted by the other factors in each specific exposure scenario.

### Wild vegetable extracts altered genes expressions

Measuring gene expression is an effective approach to evaluating the underlying mechanism of cell apoptosis (41, 48). Extrinsic (e.g., *caspase-3, -8*, and *-9, Bcl-2* genes) and intrinsic (e.g., *Bax, Bcl-2, caspase-9, p53* genes) related pathways were recognized as two primary modes of apoptosis (49, 50). Specifically, *caspases* play important roles in the initiation (*caspase-8, -9*, and *-10*) and effector (*caspase-3, -6*, and *-7*) phases of apoptosis. Furthermore, the *Bcl-2* family of proto-oncogenes is the key factor to regulate apoptosis, involving the anti-apoptotic gene (*Bcl-2*) and pro-apoptotic gene (*Bax*) (49). Tumor suppressor gene *p53* controls genomic integrity, cell proliferation, and cell death through complex signaling networks. Some studies also demonstrated that *p53* is vital to induce cell apoptosis (51, 52). As important downstream genes of *p53, GADD45*α and *p21* are involved in growth arrest and apoptosis (53).

**Figure 6:**
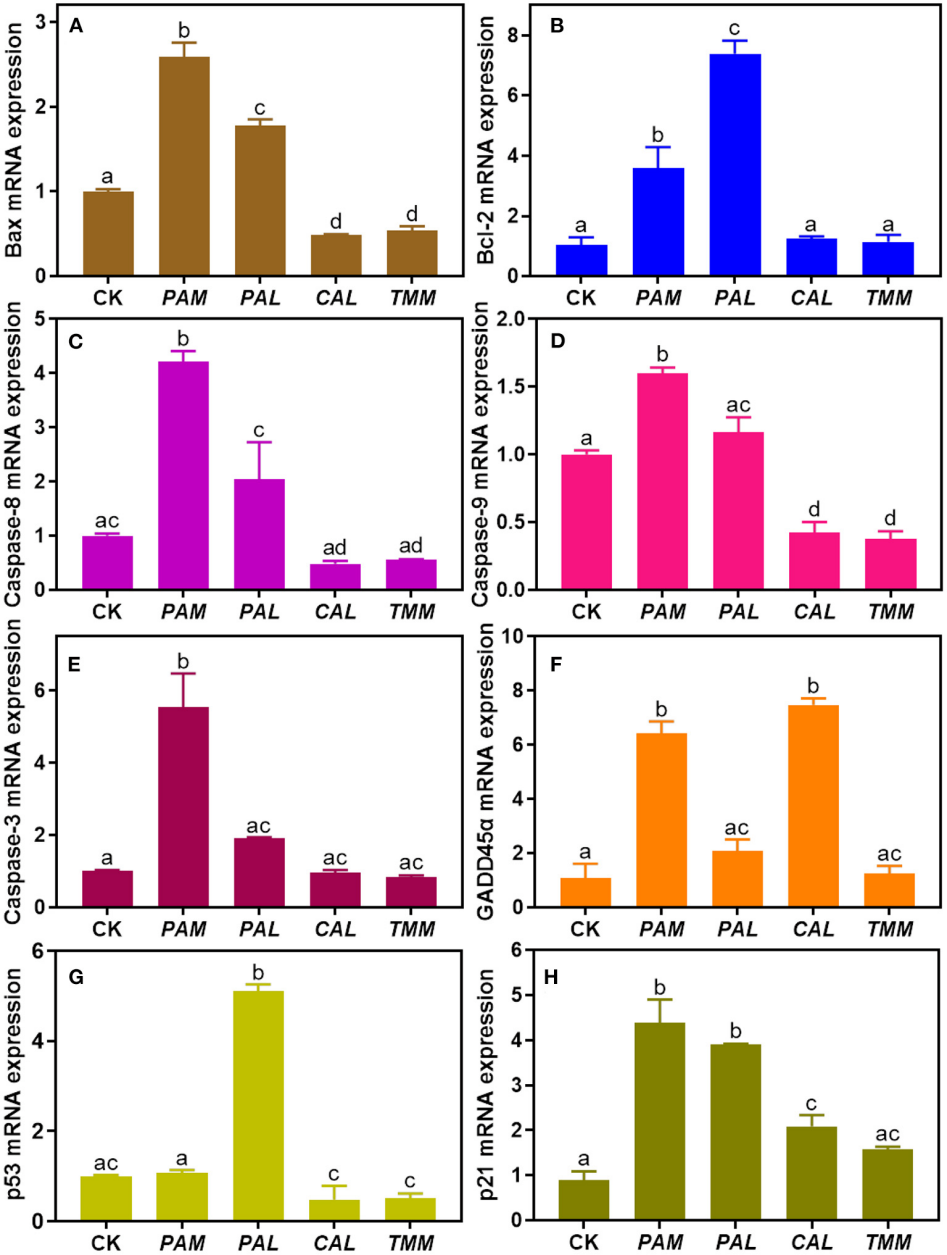
The mRNA expression levels of cell apoptosis regulatory genes (**A**: *Bax*, **B**: *Bcl-2*, **C**: *Caspase-8*, **D**: *Caspase-9*, **E**: *Caspase-3*, **F**: *GADD45*α, **G**: *p53*, and **H**: *p21*) in HGEC after being exposed to four extracts compared with the control group. The control group (CK) was treated by DMEM only. Results were represented as mean ± SD of triplicate experiments. The different letters represent significant differences at *p* < 0.05.

To further explore the underlying molecular mechanism of HGEC apoptosis induced by extracts, we assessed the genotoxicity of these extracts by measuring the alterations of regulatory gene expressions in HGEC after each exposure. The results showed that after being exposed to the *PAM* extract for 24 h, the mRNA levels of *Bax, Bcl-2, p21, GADD45*α, *Caspase-3, -8, -9* in HGEC were up-regulated from 1.6 to 6.6 folds (Figures 6A–F,H), while *p53* was maintained at the same level (Figure 6G). This result explained the observations of HGEC apoptosis under *PAM* extract exposure (Figure 4C) since the activations of *Caspase-3, -8, and*−*9* induce DNA damage and lead to cell apoptosis (15, 54). After being exposed to *CAL* extract, *p21* and *GADD45*α were significantly up-regulated (*p* < 0.05), which indicated *CAL*-induced apoptosis was probably due to the induced DNA damage pathways. In the *TMM* extract exposure group, only *p21* was up-regulated (Figure 6H), while *Bax, Caspase-9*, and *p53* were significantly down-regulated (*p* < 0.05) (Figures 6A,D,G), and the rest of the measured genes being barely altered (Figures 6B,C,E,F). The up-regulation of the *p21* gene usually causes growth arrest and promotes DNA damage, eventually leading to cell apoptosis (30). In contrast, the expression levels of all measured genes were up-regulated in the exposure group of *PAL* extract (Figures 6A–H) but did not lead to HGEC apoptosis (Figure 4D). It implicates certain chemicals in *PAL* extracts such as *PAL* polysaccharides restrained extract-induced apoptosis, which is consistent with the prior cytotoxicity results and previous studies (55, 56). In addition, Pearson correlation analyses between bioaccessible metal(loid)s in wild vegetables and gene expressions of HGEC after exposures were performed. We found bioaccessible As was positively correlated with the mRNA expression of *p53* (*r* = 0.99, *p* < 0.05) (Table 3), indicating As induced HGEC apoptosis may relate to the expression of *p53*, consistent with a previous study (57). In contrast, bioaccessible Cd was negatively correlated with the mRNA expression of *p53* (*r* = −0.96, *p* < 0.05) (Table 3), our study found that a low concentration of Cd accelerated human gastric epithelial cells proliferation, which may decrease the mRNA expression of *p53* (16).

In brief, our results showed the observed HGEC apoptosis induced by the extracts may mainly attribute to the DNA damage pathways. Furthermore, higher expression levels of *GADD45*α and *p21* genes showed DNA damage is the key pathway that induced the HGEC apoptosis under exposure to *PAM* and *CAL* extracts. However, the alterations of *GADD45*α expressions were inconsistent with the results of DNA damage, HGEC morphology, and HGEC viability under the exposures of *PAL* and *TMM* extracts, and polygenic regulation is probably the main reason for that inconsistency (43, 58). Overall, three extracts (*PAM, CAL*, and *TMM*) induced HGEC apoptosis and DNA damage as well as altered the expressions of *p53, GADD45*α, and *p21* genes. These results demonstrated DNA damage may play an important role in the observed HGEC apoptosis induced by the three extracts.

## Conclusions

In this study, Ni or Pb was predominant in all vegetable samples, with *Plantago asiatica L*. (*PAL*), *Taraxacum mongolicum* (*TMM*), *Chenopodium album L*. (*CAL*), and *Pteridium aquilinum* (*PAM*) being 13.7, 3.99, 3.24, and 1.42 mg/kg, respectively. *PAL* had the highest bioaccessible Cr, As, Cd, and Pb, while the greatest bioaccessible Ni was found in *TMM*. The *PAM, CAL*, and *TMM* extracts decreased cell viability and triggered cellular apoptosis. The bioaccessible Ni was negatively correlated with cell viability. Increased DNA damage and abnormal related mRNA expression were also observed. Interestingly, *PAL* extract with the highest bioaccessible heavy metal(loid)s did not correlate with higher cytotoxicity, indicating beneficial nutrients such as *PAL* polysaccharides may play an important role in mediating the extract-induced-cytotoxicity of *PAL*. However, it is not clear which chemical composition and cytotoxicity are causal, and only eight wild vegetable species were harvested in this study due to the season limitation, more samples from the same site or different species would be collected in our follow-up study to further validate our conclusions. Taken together, consuming wild vegetables from mining areas may cause adverse health effects on the human oral cavity. Both oral bioaccessibility and cytotoxicity data should be considered for accurate human risk assessments.

## Data availability statement

The original contributions presented in the study are included in the article/supplementary material, further inquiries can be directed to the corresponding author/s.

## Author contributions

WT: conceptualization, investigation, formal analysis, and writing—original draft. PG: investigation, conceptualization, formal analysis, and writing—review and editing. D-PZ, M-YZ, C-CW, and J-JL: investigation, validation, and formal analysis. Z-XW and J-MW: data analysis. Y-YN: supervision and conceptualization. PX: project administration, supervision, conceptualization, funding acquisition, and writing—review and editing. All authors contributed to the article and approved the submitted version.

## Funding

This work was supported in part by the Yunnan Agricultural Basic Research Joint Special Project (202101BD070001-023), the National Natural Science Foundation of China (41967026 and 21906134), the Yunnan Innovative Research Team (202005AE160017), the Natural Science Foundation of Hunan Province (2021JJ30479), Top Young Talents Project of National Forestry and Grassland Administration (2020132613), the Yunnan Fundamental Research Projects (2019FB014), the Yunnan Thousand Youth Talent Program (YNQR-QNRC-2018-049), the Open Project of Beijing Key Laboratory of Toxicological Research and Risk Assessment for Food Safety (KF-2020–01), and the Research Foundation of Yunnan Education Department (2022J0508, 2021Y231, and 2021Y237).

## Conflict of interest

The authors declare that the research was conducted in the absence of any commercial or financial relationships that could be construed as a potential conflict of interest.

## Publisher's note

All claims expressed in this article are solely those of the authors and do not necessarily represent those of their affiliated organizations, or those of the publisher, the editors and the reviewers. Any product that may be evaluated in this article, or claim that may be made by its manufacturer, is not guaranteed or endorsed by the publisher.
